# Cognitive and Behavioral Inhibition Deficits in Parkinson’s Disease: The Hayling Test as a Reliable Marker

**DOI:** 10.3389/fnagi.2020.621603

**Published:** 2021-01-15

**Authors:** Antònia Siquier, Pilar Andrés

**Affiliations:** ^1^Neuropsychology and Cognition Research Group, Department of Psychology, Research Institute of Health Sciences (IUNICS), University of the Balearic Islands, Palma, Spain; ^2^Balearic Islands Health Research Institute (IdISBa), Palma, Spain

**Keywords:** Parkinson’s disease, inhibition, cognitive flexibility, neuropsychology, impulsivity, executive functioning, Hayling test, ecological validity

## Abstract

**Objective:**

The present study seeks to provide an overview of executive (inhibition and flexibility) deficits in Parkinson’s disease (PD) by combining a cognitive and behavioral approach.

**Methods:**

Fifteen PD patients and 15 healthy controls underwent a neuropsychological and behavioral assessment including the Hayling and Trails Tests, the Questionnaire for Impulsive–Compulsive Disorders in Parkinson’s Disease (QUIP-RS), the Behavior Rating Inventory of Executive Function (BRIEF-A), and the Short Form-36 Health Survey (SF-36). The level of awareness of executive functioning was also analyzed. We finally explored how these neuropsychological and clinical outcomes could relate to each other.

**Results:**

PD patients performed significantly worse in both neuropsychological tasks designed to evaluate inhibition abilities. They also reported more inhibition difficulties in everyday life and poorer quality of life. Associations between neuropsychological measures and self-reports were found. Moreover, as indicated by the discrepancy score, PD patients were as accurate as their relatives in self-reporting their executive daily difficulties.

**Conclusion:**

Inhibition and cognitive flexibility impairments assessed by the neuropsychological tests (Hayling and Trails tests) seem to capture daily life executive problems in PD. Furthermore, our study provides a deeper understanding of PD patients’ and their relatives’ experience of these executive dysfunctions.

## Introduction

Inhibitory control is one of the hallmark executive processes impaired in Parkinson’s disease (PD) (Dirnberger and Jahanshahi, 2013). It involves the ability to control one’s attention, behavior, thoughts, and/or emotions to overcome pre-potent or inadequate responses (Diamond, 2013). Such deficits may have severe negative effects on quality of life in patients and their caregivers (Kudlicka et al., 2014; Monchi et al., 2016). They may also result in impulsivity or lack of flexibility, arising as risk factors to develop Impulse Control Disorders (ICD) (Vitale et al., 2011; MacDonald and Byblow, 2015; Santangelo et al., 2017), which encompasses a wide range of behaviors such as compulsive gambling, buying, sexual behavior, and eating and related disorders (including hobbyism and punding; Weintraub and Claassen, 2017). Thereby, the assessment of inhibition provides a relevant cognitive target to identify individuals at risk of developing ICD by using tests that are sensitive.

Neuroimaging studies have suggested that inhibitory control relies on the prefrontal cortex and basal ganglia, which degenerate in PD (Kim and Lee, 2012). The Hayling Test was precisely conceived to assess inhibitory difficulties in patients with frontal damage (Burgess and Shallice, 1996; Andrés, 2001) and has proven to be especially sensitive to detect early inhibitory impairment in several neurodegenerative diseases, mainly in Frontotemporal dementia (FTD) and Alzheimer’s disease (AD) (Hornberger et al., 2010; Matías-Guiu et al., 2019; Vestberg et al., 2019). In line with the neurocognitive paradigm, it has been shown that impulsivity in PD can also be observed as a difficulty with response-inhibition, which neural substrate is linked to a fronto-striatal network disruption (Palermo et al., 2017a). Particularly, in two major functional areas innervated by the Subthalamic Nucleus (STN): the anterior cingulate cortex (ACC; Palermo et al., 2017a, 2018) and the orbitofrontal cortex OFC (Zgaljardic et al., 2006; Palermo et al., 2017b). Overall, these results seem to suggest an overlapping of neural networks involved in behavioral and cognitive inhibition.

Recently, Foley et al. (2019) compared inhibition performance in 18 patients with PD on three cognitive tasks: the Stroop and Hayling Tests and the Elevator Counting with Distraction subtest from the Test of Everyday Attention. Interestingly, although PD patients demonstrated lower scores on all tests, only the Hayling Test was sensitive enough to detect significant inhibition deficits in PD. Their findings lead to suggest that these tasks measure different inhibitory processes and that the suppression of dominant responses assessed by the Hayling test might be the most sensitive type of inhibition in PD. Two additional studies have previously shown differences in the Hayling test between PD patients and healthy controls (O’Callaghan et al., 2013; Bayard et al., 2017).

Cognitive flexibility, linked to inhibition, has also been found impaired in PD. The Trail Making Test (TMT, Reitan, 1992) has been commonly used to measure these deficits (Kudlicka et al., 2011), and it has been shown that it contributes to the prediction of daily functioning in PD (Higginson et al., 2013). Moreover, the Trail Making Test part B has been identified as one of the most accurate test to target PD patients with higher risk to develop PD-MCI (Biundo et al., 2013) and dementia (De Roy et al., 2020). Nevertheless, this traditional measure has some limited utility because of the effects of age, level of education and cultural differences, that may bias the results. Furthermore, given the multiple cognitive domains that TMT requires (e.g., cognitive flexibility, speed processing, inhibitory control, visual working memory/sequencing, and set-switching, Fellows et al., 2017), it is not surprising that it is sensitive to neurological dysfunction. Related to this, neuroimaging studies generally identified several prefrontal and parietal networks in mediating TMT performance (Varjacic et al., 2018). In that sense, Chan et al. (2015) argued that its capacity for detecting frontal executive dysfunction appears rather limited.

To overcome the shortcomings of the TMT, Portellano and Martínez Arias (2014), introduced the Trails Test or TESen (in Spanish). It is based on the traditional TMT, but it uses colors and forms instead of letters, minimizing the influence of language and allowing broader application for individuals with low educational level and cross-cultural studies. It comprises four different conditions (trails) of increasing difficulty that are administered consecutively to assess different executive components (e.g., inhibition, working memory and cognitive flexibility). Therefore, the inclusion of new conditions in contrast to the 2 included in the TMT offers greater versatility and allows to cover a broader number of executive processes, making it more sensitive and enabling the identification of more executive vulnerabilities. At the same time, it provides derived executive index (i.e., number of errors, Accuracy, and Total executive score) to better isolate executive control cognitive processes that underly TMT-B performance.

Given the multidimensional and complex nature of inhibition control, it is important to consider different executive processes impaired in PD. Moreover, recent authors argued that the low ecological validity of most traditional neuropsychological tests may not fully capture the specific problems with EF in patients’ day-to-day functioning (Koerts et al., 2011; Kudlicka et al., 2014; van der Linden et al., 2019). By contrast, self and caregiver’s questionnaires would capture the real-life symptomatology of these patients, due to its higher ecological validity (Isquith et al., 2013; Mariano et al., 2020).

Attending to these arguments, the Behavior Rating Inventory of Executive Function–Adult Version (BRIEF–A; Roth et al., 2005), a self- and informant questionnaire that provides an overview of an adult’s executive functions or self-regulation in his/her everyday environment and has been applied in a wide range of clinical conditions such as traumatic brain injury, mild cognitive impairment and attention deficit disorder would be a useful tool in PD.

Despite the prevalence of executive dysfunction, impulsivity and cognitive rigidity and their impact on patients’ and caregivers’ quality of life (Martínez-Martín et al., 2007; Leroi et al., 2012; Lawson et al., 2016), their relationship is complex and remains a matter of debate. Some studies have combined objective and subjective approaches to measure specific EF difficulties in PD and have explored how they relate to each other (Koerts et al., 2011; Kudlicka et al., 2013; Lanni et al., 2014; Vlagsma et al., 2017). Surprisingly, while it has been commonly found that subjective cognitive complaints (namely focused on memory deficits) are related to poorer cognitive performances (Mills et al., 2016; Hong et al., 2018), a consistent finding in the above previous studies is the lack of significant associations between objective and subjective measures of EF. These findings lead to the conclusion that both approaches contribute in a different level to the assessment of EF in PD patients (Løvstad et al., 2016), but also raise the issue of awareness of executive functioning (Kudlicka et al., 2013; Vlagsma et al., 2017) in these patients. From a neurocognitive approach, prefrontal cortex impairment is considered the main hub to explain a reduction in self-awareness (O’Keeffe et al., 2007). In particular, a recent study points out that fronto-striatal and cingulo-frontal dysfunction may reflect impairment in metacognitive-executive abilities (including response-inhibition, action monitoring and error awareness) and promote compulsive repetition of behavior (Palermo et al., 2017b).

In summary, the aim of the present study was to explore the extent to which inhibition and cognitive flexibility are affected in non-demented PD patients and reverberate in daily life, by combining performance-based measures with self and informant questionnaires of executive functions, impulsivity and QoL. We also examine how the patient’s and their relatives’ subjective experience of EF difficulties in daily life are related, based on a discrepancy score, a measure of the level of agreement between both assessments.

Our hypothesis was that PD patients would perform worse than the healthy controls in neuropsychological tests. We also predicted that they would report greater impulsivity alterations and executive difficulties in daily functioning in comparison with healthy participants and that these two dimensions of executive functions would be related. Finally, we were interested in exploring the level of agreement between self- and career-reported executive abilities in patients with PD, as a way to look into patients’ awareness of executive functioning.

## Materials and Methods

The study was carried out in accordance with the ethical guidelines set in the Declaration of Helsinki (1964), with the approval of the local ethics committee. All participants provided informed consent before participation.

Patients and controls underwent a comprehensive neuropsychological and clinical assessment, during the on phase, that was performed in a single session lasting approximately an hour and a half. Prior to this assessment, the diagnostic evaluation was based on the guidelines of the Task Force commissioned by the Movement Disorder Society to identify Mild Cognitive Impairment (Litvan et al., 2011). To capture the whole spectrum of cognitive functions impaired, PD patients were assessed with the Parkinson’s Disease-Cognitive Rating Scale (PD-CRS; Pagonabarraga et al., 2008) by the neuropsychologist, an PD-specific cognitive scale which is recommended by the Movement Disorder Society Task Force to identify PD-MCI-Level I, at several stages of the disease.

### Participants

Fifteen PD patients (one woman, age range 45–80) who consulted to a Department of Neurology of a tertiary hospital in Mallorca (Spain) were recruited. All patients fulfilled the UK Brain Bank diagnostic criteria for PD. The disease severity was staged according to the Hoehn and Yahr scale (H&Y) and the Unified Parkinson’s Disease Rating Scale (UPDRS; Fahn et al., 1987), assessed by a neurologist specializing in movement disorders blind to the aim of the study. Patients in H & Y stage 4 and 5 were not included in this study. Other exclusion criteria were: (1) the presence of dementia diagnosed by a neurologist according to the Movement Disorder Society diagnostic criteria for Parkinson’s disease dementia (Dubois et al., 2007); (2) the presence of other neurological or psychiatric disorders (e.g., traumatic brain injury or schizophrenia); and (3) the presence of visual hallucinations. All patients were symptomatically stable, taking medication, and assessed during their “on” medication phase as reported by participants (see Table 1 for clinical details).

**TABLE 1:** Mean values (*SD*) for PD on demographic and clinical assessment carried out by the neurologist prior to the study.

Neurological assessment	Means (*SD*)	On-phase	Off-phase
Gender (male/female)	14/1		
PD-CRS	89.2 (12.97)		
**Disease (years):**			
Since the first symptoms appeared	6.87 (4.61)		
Since the diagnosis was made	5.56 (4.51)		
L-dopa (months of treatment)	44.4 (47.87)		
L-dopa (mg)	439.28 (268.13)		
LED (mg)	729.53 (298.23)		
UPDRS total score		25.6 (13.20)	24.6 (11.76)
Part I		0.80 (0.94)	1.86 (4.06)
Part II		6.66 (5.05)	5.80 (4.41)
Part III		17.13 (10.20)	16.8 (8.91)
Part IV		1.4 (1.33)	1.6 (1.26)
Hoehn and Yahr scale		1.77 (0.37)	1.8 (0.36)

**PD-CRS, Parkinson’s Disease-Cognitive Rating Scale; LED, total daily Levodopa equivalent dose; UPDRS, Unified Parkinson Disease Rating Scale (Fahn et al., 1987); H&Y, Hoehn and Yahr scale. For each patient, the levodopa equivalent dose (LED) was calculated following the procedures of Tomlinson et al. (2010).**

The control group was composed of 15 healthy adults (two women, 64.5 ± 4.94), recruited through advertisements. None reported a history of neurological, psychiatric relevant condition, alcohol or drug abuse, head trauma, or significant motor, visual or auditory deficits.

### Neuropsychological Assessment

The assessment included the MoCA test to detect the presence of a general cognitive deterioration. The assessment of cognitive inhibition was carried out using the Hayling test (Burgess and Shallice, 1996) in its Spanish version (Pérez-Pérez et al., 2016). This test includes two sections (A and B) containing 15 sentences each, with the final word omitted. This final word is strongly constrained by the preceding context (i.e., Cows produce…). In section A (Initiation) a simple sentence completion task must be performed (i.e., Cows produce… *milk*). In section B, however, participants must complete the sentence with a word that is unrelated to the target word and gives no sense to the sentence (i.e., Cows produce… *sky*), measuring the ability to inhibit a prepotent and overlearned response. To measure inhibitory problems, we analyzed the errors committed in Section B, as it is the most direct measure of response inhibition (O’Callaghan et al., 2013). To measure flexibility, the Trails test or *TESen* (in Spanish; Portellano and Martínez Arias, 2014) was used. For Trails 1 and 2 (non-inhibitory conditions), the respondent uses a pencil to rapidly connect circles numbered 1 through 25 forward and backward, respectively. These parts are considered simple tasks related to processing speed, assessing low-level cognitive abilities. Executive functioning intervenes more directly in Trails 3 and 4 (inhibitory conditions), where participants are asked to rapidly connect numbered circles in sequence, but alternating colors in Trails 3 and forms in Trails 4 and omitting the repeated number. Greater time to complete each task indicates poorer performance. As Trails 3 and 4 require a major variety of executive domains (such as planning, flexibility, inhibition, response fluency, or working memory), they are more sensitive to detect and identify prefrontal dorsolateral deficits.

### Clinical Assessment

Clinical assessment included the Behavior Rating Inventory of Executive Function (BRIEF; Roth et al., 2005) to examine executive function in daily life. It is a 75-item questionnaire capturing views of an adult’s executive functions or self-regulation in his or her everyday environment. There are self- and informant-report versions and answers are given on a three-point scale (i.e., never = 1, sometimes = 2, often = 3). Participants and informants are asked to assess the extent to which certain patient’s behaviors occurred during the past month.

The Spanish version of the Questionnaire for Impulsive-Compulsive Disorders in Parkinson’s Disease-Rating Scale (QUIP-RS; Weintraub et al., 2012; Sobreviela, 2015) was also administered. It is a 28-item patient-filled scale developed to measure the severity of ICD in PD. Each item is rated on a 5-point Likert scale ranging from 0 (never) to 4 (very often) assessing frequency of reported thoughts, urges/desires, and behaviors associated with ICD symptoms. The questions relate to the 4 most common inhibitory control symptoms (compulsive gambling, buying, eating, and sexual behavior), and other related disorders (hobbyism and punding as a consequence of dopaminergic medication) occurring the preceding 4 weeks. Scores for each inhibitory problem and related disorders range from 0 to 16, with a higher score indicating greater severity (i.e., frequency) of symptoms. The optimal cut off point for the combined ICDs is ≥10 and ≥7 for hobbyism-punding. Cut off points for the diagnosis for all four ICDs have been proposed with a sensitivity and specificity >80%.

Quality of life was finally measured using the Short Form-36 Health Survey (SF-36; Alonso et al., 1995), which provides a comprehensive assessment of physical, mental, and social components of participant’s health status. It is composed by two summary measures; physical and mental health scores that cover eight different dimensions of health-related quality of life (HRQoL) including physical functioning, role limitations due to physical functioning, pain, general health perception, vitality (energy and fatigue), social function, emotional role and mental health.

### Statistical Analysis

Statistical analyses were performed using the software SPSS Version 25.0. Normality was checked with Shapiro-Wilk Test and homogeneity of variances with Levene’s Test. Group differences in demographic and clinical characteristics were analyzed with independent two-tailed *t*-tests and analyses of variance (ANOVA) for normally distributed continuous variables, or Mann-Whitney *U*-test for non-normally distributed ones. In addition, Spearman rho correlations between cognitive measures of inhibition (Hayling and Trails test) and clinical variables (QUIP-RS, BRIEF-A, SF-36) were also calculated. A *p*< 0.05 was considered statistically significant.

## Results

Demographic and clinical characteristics are reported in Table 2. PD patients and controls did not differ significantly with respect to age, education, gender distribution or global cognition (MoCA).

**TABLE 2:** Mean (*SD*) values on demographics, clinical and general cognitive data (mean of raw scores and SDs), from PD patients and controls; *p*-values and effect sizes are provided.

Variable	PD	Controls	t/U	*p*-value	*d*
Age	67.3 (9.7)	67.1 (5.64)	–0.29	0.93	0.034
Years of schooling	13.4 (4.6)	14.1(3.08	–0.38	0.64	0.173
MoCA test^a^	26.5 (2.4)	27.6 (1.2)	–1.2	0.14	0.561
QUIP-RS	2.07 (2.53)	0.53 (0.74)	2.26	0.032	0.839
SF-36 physical health	64.27 (22.75)	80.13 (6.32)	39.5	0.015^§^	–0.649
SF-36 mental health	59 (9.61)	77.53 (7.48)	14	0.001^§^	–0.876

**Comparison between PD and control groups (t/U-test significance); PD, Parkinson’s disease; MoCA, Montreal Cognitive Assessment; MoCA maximum score, 30; cut-score ≥ 26; QUIP-RS, Questionnaire for Impulsive-Compulsive Disorders in Parkinson’s Disease-Rating Scale; SF-36, Short Form-36 Health Survey. ^§^ Mann–Whitney test used. The rest were independent samples t-tests. For the Mann–Whitney test, effect size is given by the rank biserial correlation. ^*a*^Looking into individual performance, 6 patients were just below the cut off score for mild cognitive impairment (MoCA cut off score = 26): four of these patients’ score was 25 and two patients’ was 22.**

### Neuropsychological Scores

As expected, PD patients performed worse than control participants on both tasks designed to evaluate inhibition abilities. Tables 3, 4 provide data from these two inhibitory tests for the two groups.

**TABLE 3:** Neuropsychological performance on Hayling for PD patients in on-phase of the disease and healthy controls.

			PD	% PD	Controls	*%* HC	*p*-value
Hayling test	Total number of errors	A: initiation condition	0.266 (0.593)	1.17 (3.96)	0.00 (00)	0 (0)	0.164
		B: inhibition condition	12.53 (3.85)	83.56 (25.68)	4.13 (3.27)	27.56 (21.8)	0.001
	Type of response	Type 1	1.13 (2.16)	7.56 (14.45)	0.00 (0.00)	0 (0)	0.052
		Type 2	10.26 (2.86)	68.44 (19.1)	4.13 (3.27)	27.56 (21.8)	0.001
		Type 3 (correct response)	3.6 (2.61)	24 (17.42)	10.8 (3.32)	72 (22.14)	0.001

**Means and standard deviations (in brackets) for type of errors (raw scores and percentages). P-values are provided for raw scores. Percentage of type of errors and correct responses was calculated as the proportion of errors or correct responses from the total sentences to complete in each section (15).**

**TABLE 4:** Neuropsychological performance on Trails Test for PD patients and healthy controls.

		PD	Controls	*p*-value
Trails test	Speed non-inhibition condition (Trails 1 and 2)	322.40 (192.84)	187.2 (51.48)	0.033
	Speed inhibition condition (Trails 3 and 4)	472.2 (238.13)	246.06 (56.53)	0.003
	Inhibition condition—Errors	1.6 (1.35)	0.30 (0.48)	0.004
	Inhibition condition—Accuracy	95.46 (3.97)	99.09 (1.32)	0.004
	Inhibition condition—Total executive score	9.4 (4.83)	15.91 (3.54)	0.001

**Means and standard deviations (in brackets) of type of errors (raw scores) and time to complete the task (in seconds) are provided. Accuracy, executive global score and p-values are also provided. Accuracy formula; Acc = (correct–incorrect/correct) × 100; Total Executive Score: E_*total*_ = (correct–incorrect/speed) × 100.**

First, total number of errors were analyzed in the Hayling test. A higher scoring in section B suggests that participants are less capable to suppress an automatic response and, thus, suggests an inhibitory impairment. A univariate 2 (group) × 2 (section A vs. B) ANOVA revealed a significant effect of group [*F*(1, 28) = 43.32, *p* < 0.001, η*_*p*_*^2^ = 0.607] and section [*F*(1, 28) = 156.6, *p* = < 0.001, η_*p*_^2^ = 0.848]. The interaction between these two factors was also significant [*F*(1, 28) = 38.517, *p* = < 0.01, η*_*p*_*^2^ = 0.579], indicating that the difference between groups was greater for section B. This was confirmed by *post hoc* comparisons (Tukey HSD test), showing that the total number of errors was similar between PD participants and controls for section A [*t*(28) = −0.28, *p* = 0.992, *d* = −0.052], but not for section B, where control participants exhibited a significant better inhibitory performance [*t*(28) = 9.04, *p* = < 0.001, *d* = 1.65].

In addition, errors in section B were classified into one of the following three possible categories. Type 1 responses that were actual completion-responses received an error score of 3. Type 2 responses that were semantically connected to the sentence in some way received an error score of 1. Type 3 responses, unrelated to the sentence, as required by the task instructions, received an error score of 0.

**FIGURE 1:**
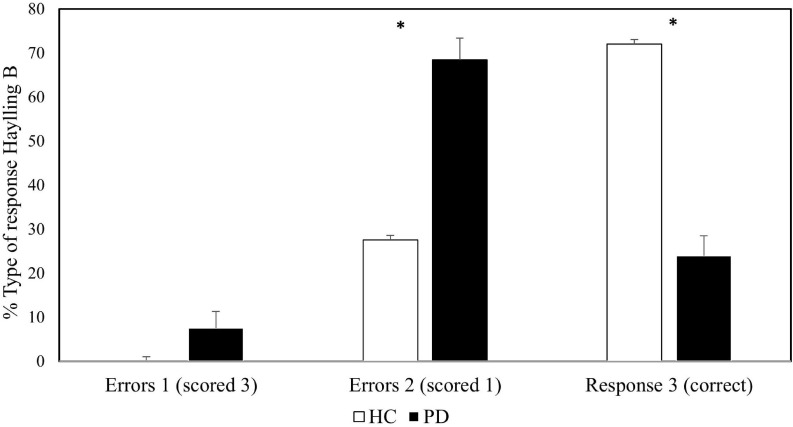
Proportion of type of response on the inhibitory section of Hayling Sentence Completion Test across groups. Error bars represent standard errors.

Under these conditions, a univariate 2 (group) × 3 (Type of response) ANOVA revealed a significant type of response main effect [*F*(1, 28) = 102.06, *p* < 0.001, η*_*p*_*^2^ = 0.785], and a significant interaction between type of response and group [*F*(1, 28) = 40.27, *p* < 0.001, η*_*p*_*^2^ = 0.59]. However, the effect of group was not significant [*F*(1, 28) = 1, *p* = 0.326, η*_*p*_*^2^ = 0.034]. The interaction showed that patients committed more Type 2 errors [*t*(28) = 5.48, *p* = < 0.001, *d* = 1.19], and fewer correct Type 3 responses [*t*(28) = 6.59, *p* = < 0.001, *d* = 2.41], than healthy participants. PD patients also showed a trend to commit more Type 1 errors [*t*(28) = 2.03, *p* = 0.052, *d* = 0.74] (see Figure 1).

For the TESen test, two completion times were calculated by averaging standard scores on Trails 1 and 2 (non-inhibitory condition) and Trails 3 and 4 (inhibitory condition). Number of “no switching” errors, accuracy [*Acc* = (correct–incorrect/correct) × 100] and a total execution score [*E*_*total*_ = (correct–incorrect/speed) × 100] were also calculated for the inhibition condition.

**FIGURE 2:**
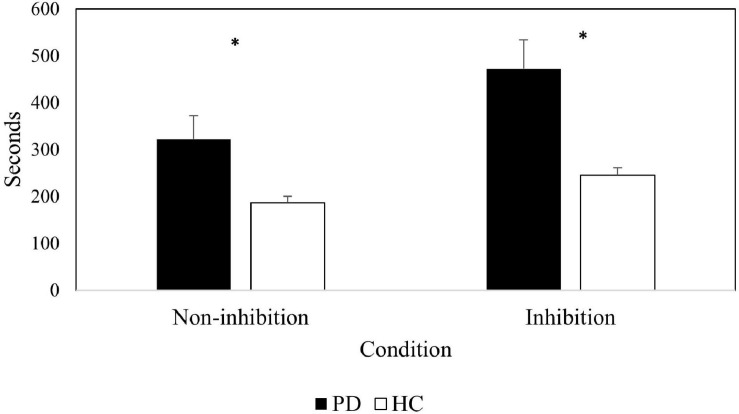
Completion time in seconds for patients and controls in both conditions of the Trails test: non-inhibitory and inhibitory. Error bars represent standard errors.

Results on the Trails test are presented in Table 4. A 2 (group) × 2 (non-inhibitory/inhibitory conditions) repeated measures ANOVA on completion times revealed a significant main effect of group [*F*(1, 28) = 10.56, *p* < 0.01, η*_*p*_*^2^ = 0.273], as well as a significant effect of condition [*F*(1, 28) = 46.68, *p* < 0.001, η*_*p*_*^2^ = 0.625], whereby the inhibition condition yielded longer completion times. A significant group × condition interaction [*F*(1, 28) = 8.87, *p* < 0.01, η*_*p*_*^2^ = 0.24] was also found. We further examined this interaction (see Figure 2) by conducting Mann-Whitney tests. Results revealed significant differences between groups in the inhibition condition (*U* = 192, *p* = 0.001) as well as in the non-inhibition condition (*U* = 164.5, *p* = 0.033), which difference between groups was greater for the inhibition conditions. We also observed a significant effect of group on the number of inhibition errors (“no switching” behavior) (*U* = 180, *p* = 0.003), indicating that PD patients committed a higher number of errors as compared to the control group. In addition, accuracy (*U* = 45, *p* = 0.004) and executive total scores (*U* = 180, *p* = 0.001) were also significantly different between groups.

Also, paired *t*-test comparisons within groups revealed differences in completion task time among conditions in healthy controls [*t*(14) = 7.11, *p* = 0.001, *d* = 1.84] as well as in PD patients [*t*(14) = 5.09, *p* = 0.001, *d* = 1.32], indicating a similar pattern in performance between groups.

### Clinical Scores

The scores in the BRIEF-A are composed by two main indexes: Behavioral Regulation (BRI*)* and Metacognition (MI*)*, which yield a composite index score, the Global Executive Composite (*GEC*). The BRI index contains four subscales (i.e., Inhibit, Shift, Emotional Control, and Self-monitor) and reflects the ability to shift cognitive set, modulate and exert appropriate regulatory control on behavior and emotional responses. The MI index is composed by five subscales: working memory, initiation, planning, problem-solving and self-monitoring. It represents the individual’s ability to cognitively manage attention and problem solving in a variety of contexts.

The means and standard deviations of the nine clinical scales and BRI, MI, and GEC indexes for the PD patients and healthy controls are presented in Table 5. Patients reported significantly higher Global Executive Composite scores [GEC, Mann–Whitney *U* = 181, *p* = 0.004], reporting more problems in executive functions daily. Also, PD patients manifested higher behavioral regulation difficulties [BRI index, Mann–Whitney *U* = 164, *p* = 0.033],] and metacognitive problem solving [MI index, Mann–Whitney *U* = 189, *p* = 0.001]. At the subscale level, they indicated greater difficulties at all levels (inhibit, shift, self-monitor, metacognition, initiation, working memory, planning, monitoring, and organization), except for Shift and Emotional Control domains. Importantly, there were no differences between self- and informant-report for either group or index (see Table 5.1). The congruency between patients and careers reveals that PD patients had a good insight into their daily executive difficulties.

**TABLE 5:** Comparison of the BRIEF-A raw scores (Means and SDs) between (1) PD patients and healthy controls, (2) self and their corresponding relative’s.

Brief-A scales	PD	Caregiver’s	*p*-value	HC	Informant	*p*-value	*p*-value PD vs. HC
GEC	118 (27.66)	114.93 (26.22)	0.758	92.2 (15.66)	95.43 (15.98)	0.587	0.004
BRI	51.26 (11.87)	48.27 (12.33)	0.503	42 (9.29)	45.86 (11.73)	0.333	0.033
Inhibit	13.67 (3.85)	12.13 (3.20)	0.246	10.2 (1.52)	11.29 (2.37)	0.151	0.010
Shift	10.2 (3.41)	10.53 (2.85)	0.773	8.73 (2.89)	9.57 (2.53)	0.415	0.202
Emotional control	16.6 (3.89)	15.73 (5.19)	0.609	15.2 (3.67)	16.14 (5.76)	0.601	0.367
Self-monitor	10.8 (2.73)	9.87 (3.50)	0.423	7.87 (2.45)	8.86 (3.05)	0.340	0.008
MI	66.73 (16.42)	66.6 (16.31)	0.991	50.2 (7.2)	49.57 (7.74)	0.823	0.001
Initiate	12.67 (4.45)	12.33 (3.97)	0.83	9.33 (1.79)	9.64 (1.47)	0.615	0.023
Working memory	14.46 (4.3)	14.13 (3.39)	0.816	10.8 (1.93)	10.57 (2.06)	0.761	0.015
Plan/organize	15.27 (4.59)	16.0 (4.22)	0.652	11.93 (1.91)	11.92 (2.53)	0.995	0.037
Task monitor	10.13 (2.47)	10.4 (2.57)	0.775	8.13 (1.92)	7.93 (1.82)	0.771	0.041
Organization of materials	14.2 (4.43)	13.8 (4.84)	0.815	10 (2.07)	9.5 (1.99)	0.514	0.006

**p-values are provided. Parkinson’s disease; HC, healthy control; BRIEF-A, Behavior Rating Inventory of Executive Function—Adult. GEC, Global executive composite; BRI, Behavioral regulation index; MI, Metacognition index.**

**TABLE 5.1:** Comparisons of corrected discrepancy score (means and standard deviations) within-group (self- vs. informant rating) of BRIEF-A scales.

Brief-A scales	PD	HC	*p*-value
Global executive composite (GEC)	−0.02(0.27)	0.028 (0.13)	0.528
Behavioral regulation index (BRI)	−0.06(0.28)	0.65 (0.22)	0.198
Metacognition index (MI)	−0.0009(0.29)	−0.014(0.09)	0.867

Discrepancy scores (Kudlicka et al., 2013) were also calculated to examine the degree of agreement between self- and informant ratings on the assessment of executive functions. Contrary to the traditional analysis of simple difference scores as an index of participant-informant discrepancies, this alternative approach considers the extent of the discrepancy as well as the baseline level of scoring involved. It was calculated by subtracting the self-rating from the informant rating and dividing the difference by the mean value of the two ratings (BRIEF-A Informant-BRIEF-A Self)/[(BRIEF-A Informant + BRIEF-A Self)/2]. Discrepancy scores go from -1 to 1, where positive values indicate a self-overestimation of executive functioning ability, and negative values indicate an underestimation of self-executive abilities. Discrepancy scores close to 0 indicate close agreement between patients and informants. The BRIEF-A corrected discrepancy scores were similar in both groups, suggesting that PD patients’ assessments coincide with the ones healthy controls do of patients’ behavior (see Table 5.1). Moreover, none of these discrepancy scores were statistically different from 0 (all *p* > 0.29). These results reveal awareness of the executive functioning in our PD patients.

Results from the questionnaire for impulsive-compulsive disorders in PD (QUIP-RS) also revealed a higher frequency of self-reported inhibitory deficits in PD patients than in controls [*t*(27) = 2.26, *p* = < 0.05, *d* = 0.839]. However, despite the significant differences between groups, the ICD symptoms severity did not reach the cut-off point (≥ 10) to meet criteria for an ICD diagnosis (see Table 6).

**TABLE 6:** Spearman’s correlation coefficients for neuropsychological performance (Hayling and Trails Test), self- reported inhibition (QUIP-RS and BRIEF-A) and quality of life (SF-36).

			Trails Test			Self Brief-A			QUIP-RS	SF-36	
		Errors Hayling B	Inhibition errors	Accuracy	Executive score	GEC	BRI	MI		Mental health	Physical health
	Errors Hayling B	1	0.665**	−0.665**	−0.613**	0.572**	0.546**	0.580**	0.360*	−0.462*	−0.672**
Trails test	Inhibition errors	−	1	−1**	−0.614**	0.390*	0.386*	0.373*	0.080	–0.290	−0.422*
	Accuracy	−	−	1	0.614**	−0.390*	−0.386*	−0.373*	–0.080	0.290	0.422*
	Executive score	−	−	−	1	−0.488**	−0.463*	−0.477*	–0.085	0.613*	0.619**
Brief-A self	GEC	−	−	−	−	1	0.950**	0.961**	0.548**	–0.411	−0.735**
	BRI	−	−	−	−	−	1	0.866**	0.577**	–0.429	−0.608**
	MI	−	−	−	−	−	−	1	0.525**	–0.275	−0.753**
	QUIP-RS	−	−	−	−	−	−	−	1	–0.261	−0.477*
SF-36	Mental health	−	−	−	−	−	−	−	−	1	0.684**
	Physical health	−	−	−	−	−	−	−	−	−	1

*p < 0.05, **p < 0.01, ***p < 0.001.

Regarding quality of life (QoL), significant differences were observed in SF-36 scores between patients and controls, indicating different self-perceived mental [Mann–Whitney U = 39.5, *p* = 0.003] and physical [Mann–Whitney U = 14, *p* < 0.001] health in PD.

Finally, Spearman correlations between inhibition tasks and with other self-report/informant report questionnaires were calculated (see Table 6). First, there were strong correlations between the Hayling (errors in section B) and the TESen tests, adding validity to these tests as instruments to measure inhibitory functions. Second, errors in the Hayling test correlated with executive difficulties in everyday life and health. For example, they correlated with difficulties in impulse control (QUIP-RS; *r* = 0.360, *p* < 0.05), self-report daily executive problems (GEC: *r* = 0.572, *p* < 0.001), behavior regulation dysfunctions (BRI: *r* = 0.546, *p* < 0.01) and metacognition difficulties (MI: *r* = 0.580, *p* < 0.01). They also correlated with poor mental (SF-36: *r* = −0.462, *p* < 0.05) and physical health (SF-36: *r* = −0.672, *p* < 0.001). These results suggest that inhibition errors may be considered as a good indicator of executive deficits at different levels.

**FIGURE 3:**
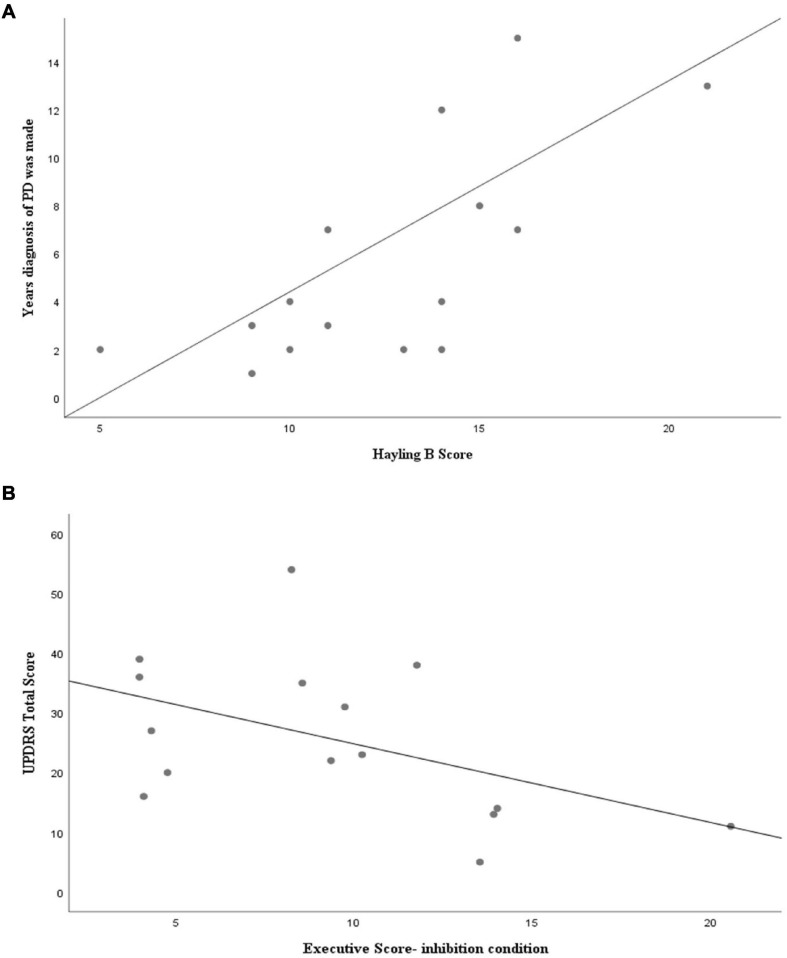
Spearman’s correlation coefficients for **(A)** patients’ Hayling B performance and years since PD diagnosis was made (*R* = 0.739; *p* = 0.002) and for **(B)** UPDRS Total Score and Trails executive total score in the inhibition condition (*R* = −0.593; *p* = 0.002).

Correlations between pathology (UPDRS-Total Score and years since diagnosis of PD was made) and cognitive performance were also calculated. Figure 3 presents the highest correlations obtained, showing the positive correlation between years since PD diagnosis and errors in Hayling B, and the negative correlation between UPDRS and performance on the inhibition condition in the TESen test.

## Discussion

There is an urgent need to understand the everyday effects of inhibitory dysfunction and its behavioral manifestations in PD to provide appropriate support for patients and their families. The objective of this study was to examine inhibition and cognitive flexibility in PD from a cognitive and behavioral perspective. To do this, we included neuropsychological tests, self-administered questionnaires, and caregivers’ evaluations. We also explored how these variables could relate to each other and to different dimensions of everyday life.

Following previous literature, we expected significant differences between PD patients and controls in inhibition. From a neuropsychological perspective, the existence of significant differences between PD patients and controls in Hayling and Trails tests corroborate inhibition difficulties in these patients. This poor performance is consistent with previous studies (Obeso et al., 2011; O’Callaghan et al., 2013; Martyr et al., 2019).

Recent neuroimaging studies converge in showing a relation between Hayling test and almost all regions of the frontal lobe, mainly involving the orbitofrontal cortex (OFC), suggesting that a network of orbitofrontal regions is involved in the performance of this task (O’Callaghan et al., 2013; Kobayakawa et al., 2017). These findings suggest that inhibition dysfunction may relate to impairments in the orbitofrontal cortex, an area that is critical for the emotional and behavioral regulation (Hornberger et al., 2011; Poletti and Bonuccelli, 2012).

In the Trails Test, PD patients also needed more time and committed more errors than controls. Although several previous studies reported deficits on the TMT in PD (Muslimoviæ et al., 2005; Colman et al., 2009; Godefroy et al., 2010; Dirnberger and Jahanshahi, 2013; Kourtidou et al., 2015; Hessen et al., 2016; Vlagsma et al., 2017; Hoogland et al., 2018), other studies reported no difference between PD and controls (Bouquet et al., 2003; Kokubo et al., 2018), even in PD patients with ICD (MacK et al., 2013). Cools et al. (2001) have argued that impairment in task-set switching only appears when competing information is present, i.e., when the load on selection mechanisms increases. In that sense, the Trails test is more executively demanding than the TMT and it may offer greater sensitivity for the identification of a broader number of executive components affected in non-demented PD patients.

In addition, the correlations found between the neuropsychological performance in Hayling and Trails tests and clinical measures of inhibition and executive functioning suggest that these two tests could emerge as effective neuropsychological tools to assess inhibitory dysfunction in non-demented PD patients, gaining in ecological validity.

Inhibition difficulties were also reflected in self-report questionnaires (QUIP-RS). PD patients reported more behavioral manifestations of disinhibition than healthy controls and poorer mental and physical health (SF-36) (see Table 2). The prevalence of the existence of ICD symptoms seem to be in line with previous literature, which estimated the existence of incident ICD symptoms in 20% of newly diagnosed untreated PD patients (Smith et al., 2016). Related to this point, the influence of dopaminergic treatment on cognition and impulsivity behavior has been consistently reported (Weintraub et al., 2006; Weintraub and Claassen, 2017; Garcia-Ruiz, 2018). Dopamine agonists and levodopa medication may ameliorate symptoms associated with dysfunction of the motor and associative circuits, but simultaneously, can overdose the prefrontal-ventral striatal circuit and impair the functions mediated by the limbic and orbitofrontal circuits potentially inducing adverse behavioral and cognitive alterations, such as impulse control in the way to promoting pathological repetition of behavior (Ray and Strafella, 2010). However, although our patients presented significantly more ICD clinical symptoms than healthy controls, their severity did not reach the cut-off point to meet criteria for an ICD diagnosis. Hence, our patients, despite presenting some cognitive inhibitory difficulties, this did not translate into a significant difficulty to control impulsive behavior.

Looking into the impact of executive dysfunction in everyday life, as measured by the BRIEF, PD patients also reported significantly greater impairments than the controls on most of the subscales. Moreover, the pattern revealed by PD patients in executive perceived difficulties was similar to the one observed in previous studies (Koerts et al., 2011; Lanni et al., 2014; Vlagsma et al., 2017). Importantly, and in contrast to Schiehser et al.’s (2013) study, our PD patients reported the same number of problems as their relatives did, as revealed by the discrepancy score. The absence of discrepancies leads to suggest that the accuracy in self-appraisal of the executive difficulties reports in PD patients is comparable to that of the control participants as well as their caregiver’s. In this way, our findings are partially different from the study of Kudlicka et al. (2013) in which they concluded that, although PD patients with and without executive impairment were as accurate as healthy older people, PD patients without EF impairment self-reported significantly more difficulties than the dysfunctions perceived by their informants. These discrepancies may reflect awareness of internal executive difficulties that are not yet observable by the loved ones. Hence, it suggests that relative-reports could be less sensitive than self-report measures of executive function in highly functioning samples. The present study, based on the level of agreement between self- and informant-reports, suggests that PD patients had good insight into their daily life executive difficulties.

Following on the greater sensitivity of cognitive compared to behavioral inhibitory control mentioned above, it is also worth mentioned that we also observed a greater sensitivity of the flexibility measured extracted from the TeSEN than the one from the BRIEF-A (Shift score). Yet, because multiple domains of cognitive components are involved in the TeSEN test, the adoption of a different analytical approach introducing new indexes allows us to better isolate and quantify specific executive processes affected in PD. The negative associations between TESen executive indexes (i.e., number of errors, accuracy, and executive total index) and BRIEF scores (see Table 5) provide further support for these indexes as a measure of more complex executive functions. These findings suggest that executive difficulties observed in TeSen test may be indicative of everyday functional impairment. Therefore, it provides additional understanding of the specific cognitive abilities underlying TESen performance and also may have a clinical value in the sense that may contribute to increase diagnostic sensitivity and specificity. In this line, several studies have shown that the inclusion of error analysis may reveal cognitive deficits not traditionally captured using completion time (Mahurin et al., 2006; Christidi et al., 2013) and also yield increased specificity in detecting and discriminating cognitive impairment in clinical populations (Stuss et al., 2001). Nevertheless, bearing in mind the small sample size and exploratory nature of these analyses, more research is needed to confirm the utility of these indexes.

The results also revealed more interesting associations. The correlations revealed that participants who showed greater inhibition errors of Hayling B also reported more impulsivity difficulties, measured by QUIP-RS scores and Behavior Regulation Index (BRI). Similar associations between self-report executive and impulsivity difficulties and cognitive dysfunction have previously been reported (Santangelo et al., 2009; Vitale et al., 2011; Yoo et al., 2015). These correlations would suggest that the QUIP-RS and BRI scores offer some degree of accuracy, and that inhibition functioning assessed by Hayling test may overlap with behavioral manifestations of the executive difficulties elicited in these clinical questionnaires.

In addition, it appears highly clinically relevant to further investigate how inhibition deficits in PD impact the patients’ daily functioning. When exploring the relationship between both factors we found that greater errors on Hayling B correlated with poorer mental and physical health status/quality of life. In that sense, this relationship may reflect the importance of inhibition impairment in self-perception of quality of life. Our results are consistent with previous studies reporting the significant contribution of executive dysfunction on QoL and subjective health status PD patients (Klepac et al., 2008; Kudlicka et al., 2014). In this context, besides the self-reported BRIEF-A, our study adds specific neuropsychological measures, providing and accurate and holistic perspective of the impact of inhibition dysfunctions, especially as measured by the Hayling test.

This study has a possible limitation that is important to acknowledge: the small sample sizes, as is often the case in clinical samples. In a previous study, however (see Siquier and Andrés, 2020) we investigated episodic memory in a similar sample also giving rise to significant differences between patients and controls in encoding and retrieval processes. Despite the ability of our study to detect group differences in inhibition and executive functions, given the heterogeneity of PD profiles, the participants in the present study may not be representative of the broader PD population. Thus, further investigations including larger sample sizes would be desirable to increase the chances to investigate further the pattern of executive deficits and the relationship between cognitive variables and clinical measures in early PD.

Besides the above-mentioned constraints, the assessment of inhibitory abilities from a neuropsychological and behavioral perspective brings an interesting combination of measures and allows to investigate impulsive behavior in PD from different angles. Furthermore, this approach provides a deeper understanding of PD patients’ metacognitive skills and explores the unexpressed needs of patients, making it possible to provide appropriate support and improve therapeutic treatments. Our results confirm the presence of inhibitory dysfunction in non-demented PD, and extend the results reported in previous studies of inhibition impairment adding self and reported clinical measures. They also reveal the Hayling test as a sensitive tool to detect inhibitory deficits in PD patients and a good indicator of quality of life in this clinical population. Hence, it may help to disentangle the nature of the inhibition control, the perception of this impairment and how it impacts in patients’ quality of life. However, given the complexity and the multidimensional nature of executive functions, further studies should investigate the relationship between different executive and inhibitory process (for example, controlled and automatic) and others clinical features that would play a crucial role on their everyday life before firmer conclusions can be reached.

## Data Availability Statement

The raw data supporting the conclusions of this article will be made available by the authors, without undue reservation, to any qualified researcher.

## Ethics Statement

The studies involving human participants were reviewed and approved by the Comité d’ètica d’investigació de les Illes Balears. The patients/participants provided their written informed consent to participate in this study.

## Author Contributions

AS conducted the literature search, material preparation, data collection, and analysis. PA was involved in writing-review, editing and supervision. Both authors contributed on the first draft of the manuscript writing and read and approved the submitted manuscript.

## Conflict of Interest

The authors declare that the research was conducted in the absence of any commercial or financial relationships that could be construed as a potential conflict of interest.
